# *‘Why would we not want to keep everybody safe?’* The views of family members of people who use drugs on the implementation of drug consumption rooms in Scotland

**DOI:** 10.1186/s12954-022-00679-5

**Published:** 2022-08-29

**Authors:** Tessa Parkes, Tracey Price, Rebecca Foster, Kirsten M. A. Trayner, Harry R. Sumnall, Wulf Livingston, Andy Perkins, Beth Cairns, Josh Dumbrell, James Nicholls

**Affiliations:** 1 Salvation Army Centre for Addiction Services and Research, Faculty of Social Sciences, University of Stirling, Stirling, Scotland, UK; 2 School of Health and Life Sciences, Glasgow Caledonian University, Glasgow, Scotland, UK; 3 Liverpool John Moores University, Public Health Institute, Liverpool, Scotland, UK; 4 Faculty of Social Sciences, Glyndwr University, Wrexham, Wales, UK; 5 Figure 8 Consultancy Ltd, Dundee, Scotland, UK; 6 Faculty of Health Sciences and Sport, University of Stirling, Stirling, Scotland, UK

**Keywords:** Drug consumption rooms, Safer injection sites, Supervised injection facilities, Harm reduction, Overdose prevention, Lived experience, Problem drug use, Families, Qualitative research, Scotland

## Abstract

**Background:**

People who use drugs in Scotland are currently experiencing disproportionately high rates of drug-related deaths. Drug consumption rooms (DCRs) are harm reduction services that offer a safe, hygienic environment where pre-obtained drugs can be consumed under supervision. The aim of this research was to explore family member perspectives on DCR implementation in Scotland in order to inform national policy.

**Methods:**

Scotland-based family members of people who were currently or formerly using drugs were invited to take part in semi-structured interviews to share views on DCRs. An inclusive approach to ‘family’ was taken, and family members were recruited via local and national networks. A convenience sample of 13 family members were recruited and interviews conducted, audio-recorded, transcribed, and analysed thematically using the Structured Framework Technique.

**Results:**

Family members demonstrated varying levels of understanding regarding the existence, role, and function of DCRs. While some expressed concern that DCRs would not prevent continued drug use, all participants were in favour of DCR implementation due to a belief that DCRs could reduce harm, including saving lives, and facilitate future recovery from drug use. Participants highlighted challenges faced by people who use drugs in accessing treatment/services that could meet their needs. They identified that accessible and welcoming DCRs led by trusting and non-judgemental staff could help to meet unmet needs, including signposting to other services. Family members viewed DCRs as safe environments and highlighted how the existence of DCRs could reduce the constant worry that they had of risk of harm to their loved ones. Finally, family members emphasised the challenge of stigma associated with drug use. They believed that introduction of DCRs would help to reduce stigma and provide a signal that people who use drugs deserve safety and care.

**Conclusions:**

Reporting the experience and views of family members makes a novel and valuable contribution to ongoing public debates surrounding DCRs. Their views can be used to inform the implementation of DCRs in Scotland but also relate well to the development of wider responses to drug-related harm and reduction of stigma experienced by people who use drugs in Scotland and beyond.

**Supplementary Information:**

The online version contains supplementary material available at 10.1186/s12954-022-00679-5.

## Background

### Drug consumption rooms and the potential for the reduction of drugs harms in Scotland

Globally, an estimated 500,000 people lost their lives to drug-related death in 2020–2021 [1]. In the UK, drug-related deaths (DRDs) represent a significant public health crisis [2, 3]. Scotland (UK) is currently experiencing 30.8 DRDs per 100,000 of the population, which is three times higher than the UK as a whole and one of the highest rates in Europe [4]. Furthermore, despite provision of a range of harm reduction initiatives (e.g. needle and syringe programmes), a large outbreak of human immunodeficiency virus (HIV) is currently ongoing among people who inject drugs in the city of Glasgow, where HIV prevalence rose more than tenfold between 2011 and 2018 [5].

Alongside a wide range of initiatives to address such drug-related harms in Scotland has been a call for the introduction of drug consumption rooms (DCRs) [6]. DCRs are legally sanctioned, accessible, low-threshold services that aim to offer a safe, hygienic environment for people to consume pre-obtained drugs under supervision [7–9]. Many names are used to describe DCRs, including safe injecting sites/centres, supervised injecting facilities, and overdose prevention sites/centres. There are over 200 DCRs now in operation across the world [7–10].

DCRs are a public health intervention designed to reduce overdose morbidity and mortality, risk behaviours (i.e. sharing of injecting equipment) associated with blood-borne virus (BBV) transmission and injection site infections, public injecting, and associated public disorder outcomes (such as drug-related litter), while also increasing social integration by establishing contact with, and offering harm reduction advice and services to, people who are not receiving other health and social supports [11]. Some DCRs are clinical settings, while others are peer-led, community initiatives [12–14]. Some act as standalone services, while others are co-located with other provision such as medication-assisted treatment or needle and syringe exchange programmes [7]. There is evidence of a high willingness of people who inject drugs to engage with DCRs [5, 15, 16].

Available evidence, while primarily derived from a limited number of sites in Vancouver (Canada) and Sydney (Australia), suggests that frequent DCR use among people who use drugs may increase engagement with treatment services [7, 17, 18]. A study of a Vancouver DCR demonstrated a 35% reduction in fatal overdose in the surrounding area (500 m) [19]. A more recent study found a reduction in all-cause mortality for those who attended the same site on a weekly basis [20]. A six-year cohort study in the USA exploring the impacts of an unsanctioned DCR found that use of this facility was associated with a reduction in emergency department visits and hospitalisation relating to drug use [21]. By their very nature, DCRs respond to significant numbers of overdose events and, critically, to date there have been no recorded overdose deaths in a DCR anywhere in the world. However, measuring the precise impact of DCRs on overall DRDs is difficult due to a wide range of confounders, and the current lack of trial data remains a reason given by the UK Government to block their adoption [22–24]. Multi-year evaluations have found no increase in violent crime in the vicinity of DCRs, and some have found a reduction in activities such as assault and house-breaking [25–27].

### The complexities of implementing DCRs in Scotland

Pressure to introduce DCRs in Scotland has increased considerably in the last decade. However, legal barriers (real or feared/perceived) have prevented their adoption. The UK Government has opposed their introduction on the grounds that they ‘encourage’ drug use, or represent a ‘distraction’ from other interventions [23, 28, 29]. Scotland operates a devolved parliament and, while the Scottish Government has responsibility for setting health and justice policy, drug legislation is reserved to the UK Government. It is important to note that challenges to DCR implementation are not unique to Scotland and are not only related to legal issues.

The Scottish Government has repeatedly stressed their desire to implement DCRs if legislative changes were made [30–32]. A UK parliamentary inquiry of the Scottish Affairs Committee recommended that the UK Government reforms the Misuse of Drugs 1971 to allow a DCR to be established in Scotland or devolve responsibility for the Act to the Scottish Government [2]. In 2019, a Drug Deaths Taskforce (DDTF) was established by the Scottish Government to support a collective preventive response to increasing drugs deaths. It has advocated for the introduction of DCRs, either through UK-wide legislative reform or by formal devolution of powers to Scotland [33]. In 2020, an activist opened an unsanctioned mobile overdose prevention service in Glasgow city centre which operated for nine months and oversaw over 800 injections and responded to nine overdoses [34].

### Representations of DCRs and the role of personal narratives

While there has been significant support for DCRs in sections of the Scottish media, even supportive coverage (and much of the oppositional reporting) adopts potentially stigmatising language such as ‘fix rooms’, ‘shooting galleries’, or ‘junkies’ [28]. While stigma not only contributes to wider social harms, research has shown it can also create a barrier to public support for DCRs [35, 36]. The levels of public support for DCRs in Scotland have not been extensively tested: one study found that, among a representative sample of the Scottish public, support was higher in those provided with a combination of clear, factual information and personal narratives [7, 37, 38]. Prior studies have also suggested that personal narratives promote an ‘ethical consciousness’ by introducing an emotional element that counters dominant discourses and opposition [39–41]. While rarely the sole driver of policy change, the personal narratives of affected family members can play a critical role in policy advocacy. In Melbourne, Australia, family members campaigned vigorously for DCRs and won public support, despite initial political resistance. By contrast, in San Francisco this year, some family members protested at the site of a ‘linkage centre’ (not technically a DCR but essentially operating as one), expressing that, while they ‘love’ the centre, they did not want drugs to be used within the site [42]. In the UK, affected families have become prominent in advocating for drug policy reform, including DCRs [43–47]. Family member perspectives can therefore be an important vehicle for change that is often overlooked in the drugs literature [47].

In Scotland, many high-profile groups, including those bereaved through DRDs, have raised the visibility of the DCR debate [48]. Despite this, no studies have specifically explored family member perspectives or beliefs. The aim of this research was to address this knowledge gap and assess family member perspectives on DCR implementation in Scotland. The following research questions guided this study:

1. how are DCRs perceived by affected family members in Scotland?
2. what factors shape these views, and how do these shape (potential) decision-making?
3. what are the perceived barriers and facilitators to introducing DCRs, from the perspective of family members?

The data presented in this paper form part of a wider study that included exploring the views of strategic decision makers in Scotland which have been presented in a separate linked paper [49]. A further paper focusing on problem representations across the study’s two distinct datasets of family member and strategic decision makers will also follow.

## Methods

This paper is based on data from an exploratory qualitative study involving semi-structured interviews with affected family members between October 2020 and March 2021. In reporting our study methods, we have drawn upon the COREQ checklist [50] to improve transparency in describing our qualitative research processes. A specially convened lived-experience group (Experts by Experience) provided ongoing input to the whole study, acting as a feedback loop between the research team and those with personal experience. This group comprised three individuals with experience of problem drug use either as family members of people who use drugs (n = 1), or as individuals with personal experience of problem drug use (n = 2). They provided input into the study throughout, for example, by critically advising on recruitment strategy and the language used in participant materials. The overall study also benefited from the oversight of a small Research Advisory Group (RAG) representing those with particular expertise in the area of DCRs. Ethical approval was provided by the University of Stirling’s General University Ethics Panel (reference: 19/20 958).

We adopted an inclusive approach to conceptualisations of ‘family’, and defined family member as: parents, partners, sons, daughters, siblings, aunts, uncles, nieces, nephews, and grandparents, and encompassed in-law and step-relationships. While initially we had aimed to attract those who had a family member who was currently using drugs, a small number of respondents had family members who identified as being in recovery or had lost a loved one to a DRD. We considered these perspectives important to capture and expanded our study criteria with ethical approval. A convenience sampling and snowballing approach was taken to maximise the number of participants. Peer support and family groups, as well as local and national organisations located across Scotland, supported the study by circulating information and facilitating contact with associated family members. We also placed a series of advertisements on Twitter, requesting that interested parties contact researchers to express interest in participation.

All interviewees received a participant information sheet via email in advance of the scheduled interview. The participant information sheet contained information about the study and gave a brief definition of a DCR. Two female, early career researchers undertook all interviews (TP2, n = 9; RF, n = 4) using a topic guide developed by AP, RF, TP1, TP2 (see Additional file 1: Appendix 1). The guide was also reviewed and approved by JD and the Experts by Experience group. RF and TP2 had regular meetings to discuss the progress of the interviews to ensure a good level of consistency. Due to the COVID-19 pandemic, all interviews were conducted remotely, either through Microsoft Teams, Zoom, or phone. An inclusive approach was adopted to consent: participants could either provide informed written consent returned via email or verbally where consent was recorded on an audio-recorder. Interviews lasted between 45 and 70 min. All interviews were audio-recorded and transcribed. Interviewees received a £10 shopping voucher to thank them for their time and contribution to the study. Coding of interviews took place during data collection in order to inform subsequent interviews via minor changes to the topic guide.

Audio files were transcribed verbatim and coded/analysed by TP2 in NVivo (Version 12), using Spencer and Ritchie’s [51] Structured Framework Technique. Line-by-line descriptive coding was followed by gradually evolving codes into a coding framework. Once five transcripts had been coded, TP2 and RF met to discuss and develop the coding framework. Additional codes and sub-codes were added as necessary as further transcripts were coded, and discussions between RF and TP2 continued during the coding and analysis period. The memo and annotation function in NVivo 12 was used to capture reflections and links between codes and helped to inform the final coding framework. TP2 led the initial write up of the data analysis which was revised and developed further by RF in consultation with TP2 and TP1. During the process of writing up the themes, several meetings took place with the wider research team (AP, WL, JN, JD, BC, HS, KT) enabling further discussion and refinement of the thematic framework. The themes were refined further during the collaborative writing process for this paper, as is common in writing up qualitative research where writing can continue the analytical process.

## Findings

Thirteen family members living in a range of locations across Scotland were interviewed. While familial relationships included partners, siblings, and cousins, the majority were mothers, and 11 out of 13 interviewees were women. Data are presented in relation to four thematic categories: 1) awareness and knowledge of DCRs among family members; 2) facilitating use or gateways to recovery?; 3) missed opportunities and managing fear; and 4) stigma and DCRs as a paradigm shift.

### Theme one: awareness and knowledge of DCRs among family members

Interviewees had varying levels of background knowledge about DCRs. A small number of participants (n = 3) were actively involved in campaigning for drug law reform and considered DCRs to be an essential service capable of reducing DRDs, as this participant highlights:

‘I would say quite aware, like more aware probably than the general member of the public because I’ve been campaigning for over ten years for drug law reform. So I’m not an expert but I’m someone who has you know looked into all of these things because of what happened to my [family member name]’ (Participant 1, woman, cousin, West Scotland).

Those participants who had a good level of background knowledge tended to be strong advocates for DCRs. Several other participants had become aware of DCRs because of their own family member’s experiences which motivated them to find out more:

‘I pay a lot of attention to drug policy in Scotland because of the way it impacts on my own and my son’s experience’ (Participant 3, woman, mother, North Scotland).

However, others were much less aware of DCRs and had very minimal background knowledge. In such instances, the researchers gave a brief outline of what a DCR is, and what it seeks to do. This, and the information provided on the participant information sheet, meant that knowledge became developed and/or worked through as a consequence of the interviews. Those who had limited detailed knowledge of DCRs were still relatively aware of media debates surrounding DCRs. Much of this awareness related to media coverage of the unsanctioned van that was operating in Glasgow at the time of the interviews:

‘On the news, ken [you know], as I’m saying, that them getting stopped by the police and I think there is one guy in particular that is doing it in [Glasgow] on a bus’ (Participant 2, woman, mother, East Scotland).

Some of those who were strongly in support of DCRs told us that they had not always felt this way:

‘I know my son and I know what he has struggled with and I know what he is still struggling with and I know that essentially he is a very good person. Who actually needs help. So any kind of attitude I may have had in the past about setting up DCRs has drastically changed because of my lived experience’ (Participant 13, woman, mother, location unknown).

Many family members could pinpoint moments or events where they had become supportive of DCRs. Several told us that listening to people with lived experiences similar to their loved one had contributed to a shift in perspective. Often this initiated a journey of learning in which they engaged with the issue in more depth through reading and wider research:

‘It was, as soon as I started educating myself, it was like a lightbulb moment, and the DCR, the idea of a DCR was just one you know of many things that I thought “Oh my golly, I can’t believe we don’t do this as a humane society” […] But it was just, it was literally just through reading about it and thinking… immediately thinking “why on earth are we not doing this?”’ (Participant 1, woman, cousin, West Scotland).

Several had engaged with international research evidence on drug policy, not only on DCRs but related to wider reforms:

‘I’ve just been reading and then sort of other countries were trying things, you know, so Canada and Portugal were looking at radically supporting users of needle-based drugs you know, to try and reduce the harm to them. I was like “Wow that’s amazing, that’s really interesting. Yes, why would we not want to keep everybody safe?”’ (Participant 8, woman, sibling, North Scotland).

As the preceding quotations highlight, exposure to (unidentified) sources that presented positive outcomes from DCRs encouraged some family members to become strong advocates for implementation. Participants did not discuss some of the gaps and uncertainties that characterise the scientific literature on DCRs, and which have been represented in some popular news-media reporting in the UK (see Atkinson et al., 2019), suggesting that reading may have been limited to a few sources or to confirm prior beliefs. For others, listening to personal narratives was the primary motivation towards active support. In summary, within our sample there was a breadth of understanding of the role and function of DCRs. Listening to the narratives of those with first-hand experience of DCRs was particularly influential in shaping views towards them.

### Theme two: Facilitating use or gateways to recovery?

While prior research has not found evidence that DCRs encourage drug use, a small number of respondents expressed concerns that this might be a risk with regard to their loved ones [52]. This point connects with some of the concerns expressed by family members protesting in San Francisco mentioned earlier in this paper:

‘I mean, one part of me says yes, I possibly would have encouraged her to do it, but also I may not have because I’m just enabling her to take drugs’ (Participant 10, woman, mother, East Scotland).

Family members who reported concern about DCRs ‘enabling’ drug use were still supportive of implementation:

‘[…] almost all of us who are parents have engaged in enabling behaviour you know? Because we love our children, and nobody wants to see their child harming themselves even if they are an adult, so we have had to change our ways too and there is a whole education that needs to happen […] So family members need to be included in the DCR [debate], even if it’s just educating us about what it is, and what it means, and all of that is really important’ (Participant 6, woman, mother, East Scotland).

Several participants said that they believed that their loved ones had to reach ‘rock bottom’ before they would change. This term often relates to recovery concepts within the 12-step programme. While considered outdated by many critics within addiction research (e.g. [53]), the concept clearly had valence for a number of participants and framed their understanding of the role of DCRs in regard to experiences of addiction:

‘[…] one hears phrases such as reaching rock bottom before the person chooses to change, needs to reach the bottom. Needs to suffer so much pain to put their hands up and say “Oh please help”’ (Participant 9, man, relationship undisclosed, East Scotland).

Our data highlighted an underlying tension between harm reduction and abstinence-based recovery concepts which linked to this notion of ‘rock bottom’. These concerns were more prevalent among the participants who had low, or no, prior knowledge of DCRs. Most participants described DCRs as essential harm reduction, with many suggesting that, by the time a person required a DCR, they were likely to already be in a very difficult situation, akin to ‘rock bottom’:

‘I mean DCRs are really, equally as important, because once someone gets to using needles, they are seriously, you know, caught in the addiction. You know, they have reached the almost pinnacle of their addiction when they are willing to do that. Because injecting is not fun for anybody. Once someone has got to the point where they are willing to do that, they are really hooked and seriously need help’ (Participant 6, woman, mother, East Scotland).

As participant 6 outlines above, DCRs were also represented as a challenge to simplistic notions that ‘rock bottom’ was a singular and transformative event. Advocates for DCRs note that they help prevent the kind of catastrophic experiences that may be more likely to result in death than sudden transformation, though some participants saw DCRs as mitigating, rather than removing, those risks.

‘There is all different levels of rock bottom and I don’t think […] certainly someone coming into a safe consumption room is going to stop them reaching rock bottom, why are they there in the first place?’ (Participant 11, man, sibling, Central Scotland).

All family members believed that DCRs could provide a potential gateway to future recovery, although how this was conceptualised differed across the sample. For some this gateway involved creating an environment where people could inject more safely, while gradually establishing trusting relationships with non-judgemental staff. These family members believed that trusting relationships, in a predictable, stable environment, could help individuals move towards decisions to reduce drug use or seek further support:

‘If you go to a drug consumption room you are going to get suggestions and advice on where to seek treatment locally to where you live, to access the services, find out about actual recovery. Because there is (sic) thousands and thousands of drug users don’t realise that they can recover. And […] if you have never touched in with the services, or been part of anything, how would you know that you could recover?’ (Participant 4, woman, mother, East Scotland).

‘[…a DCR] lends itself to people being given just a bit of space to say “Actually I would want to do something different here….”. I think it’s the gap between what we say and what we do at a deeply compassionate level. It’s easier to blame and shame and stigmatise people, and see them as less than human, rather than see them as your son, daughter, niece, nephew, uncle, or whatever’ (Participant 3, woman, mother, North Scotland).

Although some participants saw DCRs as a gateway to recovery, the above quotations illustrate that others saw DCRs primarily as a type of enhanced harm reduction. Ultimately, whether framed in terms of ‘rock bottom’, or as an opportunity to encourage incremental change, family members who supported DCRs viewed them as a source of hope that, under their difficult circumstances, should be available as an option:

‘Hope, just absolute and utter hope, when he […] is living with you or she is living with you and she wakes up and she’s shaking and she is dreadful, you can say “Do you want me to take you, do you want me to take you to the consumption rooms […]?” So hope, that’s all you are left with in the end you know. Hope one day that they say, “I don’t want to live like this anymore”’ (Participant 7, woman, mother, North Scotland).

In summary, while some family members had concerns that DCRs might sustain drug use, they remained supportive. Some family members felt that DCRs could help facilitate future (abstinent) recovery, whereas others saw DCRs as a harm reduction intervention. Across the sample, the important role of DCRs in promoting safety was recognised.

### Theme three: missed opportunities and managing fear

Many participants expressed the view that DCRs could address some of the current gaps in the service system in Scotland. The first gap identified was a general lack of attractive services for people who inject drugs; ones that they believed would accept them and help them on their terms with less judgement. Almost all family members gave examples of situations where their loved ones had decided to seek help, but found services were not available or appropriate for their particular needs, for example, where waiting lists were unduly long, or accessing services where individuals felt they were not listened to or did not have their problems validated. One participant explained this as follows:

‘My [family member] had spoken many times over the years about him getting help and he actually had tried. He reached out to someone through the NHS [National Health Service], but then it just wasn’t facilitated very well so he then just continued what he was doing because he couldn’t get the help that he needed. So I would feel that [a DCR] would help those people’ (Participant 12, woman, partner, East Scotland).

Most participants broadly defined ‘treatment’. For many, the term treatment was taken to mean a person-centred intervention that would address underlying drivers for drug use. Several participants spoke of having been offered only one option, such as opioid replacement therapy, and felt that this had not aligned with their family member’s needs. Many suggested that a DCR could offer a valuable space where people could build trust in staff, and where there may be greater opportunities to explore diverse forms of ‘treatment’ or support:

‘Guidance and training, story sharing, events, education, all the things that happen in treatment, and by overcoming isolation and motivation to change, all these things that help and perhaps allow people […] to think about talking about the original causation’ (Participant 9, man, relationship undisclosed, East Scotland).

Several participants described a need for a person-centred, holistic approach to drug use and expressed the view that a DCR could provide this by being accessible and giving space to interpret and respond to emerging needs. Some participants discussed this in general terms, whereas others focused on specific gaps in the existing system:

‘There is no help for, not a lot of help out there […] it’s really difficult to get […] the help, and I know I’m not the only person because when I used to go along to my groups we used to talk about that’ (Participant 2, woman, mother, East Scotland).

In terms of accessibility, many interviewees highlighted the importance of DCRs being located in geographic areas of high need where people could use the service on a flexible, drop-in basis. Several felt that DCRs would help to overcome the difficulty of navigating a wide range of services:

‘There have been multiple times when my partner has gone to the GP […] they never tried to help or tried to you know give him information about programmes or anything you know, so I think I feel like just from my experience in Scotland there is sort of a gap […] it doesn’t feel that there are that many services out there and it doesn’t feel like many people know about them’ (Participant 12, woman, partner, East Scotland).

Several participants expressed that a DCR could provide a valuable, informal route into treatment and support that could potentially feel less daunting to those with drug-related problems who may have faced stigma in the past when attempting to access services. Several participants described the barriers their family members had faced when trying to access services:

‘So the first port of call for me was [his] local GP, and there was just a massive waiting list for rehabilitation and also, at that point, early on in his drug career, he wasn’t a problem. He wasn’t a problematic user, therefore didn’t fall into categories in order to get support. […] Looking back as an adult now I think “well that’s ridiculous, if somebody comes to you early doors that surely is an entry point to prevent further decline, or involvement or addiction, you know?” And now when I look back I think, and it’s one of the big things that kind of sticks with me, I think “gosh, if that had been different. My whole life could have been different if that had been different”’ (Participant 8, woman, sibling, North Scotland).

The above participant and several others suggested that having been refused support or interventions multiple times due to not meeting criteria, or there not being relevant interventions available in the area, could have the cumulative effect of causing people to distrust services and become unwilling to reach out again. A DCR was described as a way to overcome this:

‘I think there just is as many levels as possible, as many access points to treatment, support, information, the better and sometimes even just sitting in a doctor’s surgery is too much and a DCR, a drug consumption room, would be a much more informal way of accessing very formal support which doesn’t feel like you are entering a system or the system as it sometimes feels for drug users’ (Participant 8, woman, sibling, North Scotland).

DCRs were also seen as potentially easing pressure felt by families. Family members reported that, while they were constantly fearful about their loved one’s wellbeing, they could not always be their only source of support:

‘Most families like myself spend most of the time trying to figure out how to work with the person we love. There is just this fine line all the time [of] holding them with love, and offering support and help, and then having to practice tough love, and then just worrying about them all the time, about whether they are safe, what they are doing and where are they going, and are they getting drugs that are contaminated […]. And then if they are injecting or sharing needles, you know, and diseases, it’s just an ongoing nightmare of worry and concern. I think [a DCR] would create […] lower those anxiety levels for sure, for families and friends of the person who is using’ (Participant 6, woman, mother, East Scotland).

Asked about the potential impact of a DCR on their own lives, one family member commented:

‘Oh God, I think it would be massive, you know? I think the threshold of anxiety and fear that people live with when they have a family member misusing drugs is, is poorly understood. My son has been misusing drugs for seven, eight years now, and it’s really just in the last year that things have improved. So I have spent eight, nine years wondering where he is, is he alive? Not fully understanding the depth of his difficulties because we had to do a lot of work on our relationship anyway. And I think that is really poorly understood. And also the impact on his sister has been massive’ (Participant 3, woman, mother, North Scotland).

Another family member described how a DCR could have alleviated fears about her daughter during a time where she was injecting in public places:

‘They were all sharing needles and hitting each other up and oh my God it was just absolutely hellish […] whereas if there was a place like that at the time […] I would have been definitely more relaxed, definitely. I would have been able to put [my head on] my pillow at night and maybe not worry so much’ (Participant 5, woman, mother, West Scotland).

In summary, family members emphasised perceived inadequacies in current provision for people who use drugs, giving examples of long waiting times for support, and negative interactions with healthcare staff when support was able to be accessed. Family members saw DCRs as both filling gaps in current provision and establishing links between people who use drugs and access to other services such as housing.

### Theme four: stigma and DCRs as a paradigm shift

For interviewees, stigma was fundamental to the harms experienced by people who used drugs. Stigma was seen as impacting at both an individual level—by pushing individuals and their supporters away from sources of help, and at the ‘macro’ level of policy—such as delays to strategic implementation of DCRs in Scotland. Many interviewees described the impact of stigma on loved ones who had previously tried to access services. Some reported that their loved ones mistrusted addiction, treatment, and health services, and did not want to be drawn into ‘the system’ where they might be judged. For many participants, DCRs represented a first step towards a new approach to reducing stigma:

‘So (a) first of all, yes I think it would reduce the deaths. And I think it would also make people feel we are being paid attention to. We are not having to sneak away like rats into a corner to do this. We are being given a place. We are being acknowledged as people with a problem. And that is the first step in changing people’s attitude about recovery and wanting to get into recovery. As long as addicts see the establishment as anti-them, then recovery will become more threatening for them and more difficult. Surely recovery is what we want at the end of the day?’ (Participant 13, woman, mother, location unknown).

Family members also connected the stigma associated with drug use to barriers in establishing DCRs. There was significant frustration about the lack of political progress towards implementation:

‘They [Scottish Government] are way behind and there is (sic) loads of other countries that are having them. The drug figure deaths are so much lower than what the UK and Scotland are, so to me it’s common sense, it’s like I cannae [cannot] see why we shouldn’t be doing it. I really don’t see why we shouldn’t be doing it’ (Participant 4, woman, mother, North Scotland).

For one participant, stigma was the only explanation for what they felt should be a simple political decision:

‘To me it seems really, really straightforward. And so argue that the other way then: “So you want to keep drugs in the hands of criminals. You want it to be illegal and dangerous because? Could you please explain to me why that is our policy?” […] I have not heard anything, “well it’s for this very good reason that we want to keep people dying from drugs, thank you.” So why would we not flip that on its head then and be like well “why don’t we try and stop people dying from drugs?” and a really good place to start would be decriminalising use. Allowing safe consumption. Allowing access to extra services. Not making them feel like crap because they are users. Providing everything that they need in order to possibly change their life’ (Participant 6, woman, mother, East Scotland).

For many participants, the value of drug consumption rooms went beyond the individual outcomes. Rather, they viewed the introduction of DCRs as symbolic of a paradigm shift towards a health-based approach to drug problems more broadly:

‘By creating these drug consumption rooms what we are really saying is, “we have an issue as a society. And it’s our responsibility to look after these people.” And that is a big mind set shift’ (Participant 8, woman, sibling, North Scotland).

Some participants described having experienced stigma due to having a family member with drug-related problems. For example, one participant pointed out that, while she campaigns publicly for both DCRs and drug policy reform, another close family member feels unable to do so due to stigma. This participant, alongside others, perceived the introduction of DCRs as being capable of addressing multiple forms of stigma, and making it easier for families to gain community understanding of the challenges commonly experienced by people who use drugs. In this way, stigma was perceived to be a barrier to DCR introduction; yet should DCR introduction be possible, participants felt that the stigma of drug use could be minimised:

‘First and foremost, it’s keeping individuals safe who have got problematic drug use. You know, that is actually like a very small percentage of the people that actually use drugs you know daily, legal and illegal. So, you know, these people are really desperate, they are at such a low point, they are the most vulnerable people in society and it’s just I think symbolically […], it shows that we care as a society. You know, it’s just one step to showing that we should treat people who are in pain you know, which is generally what it is, with more compassion. […] I would hope that you know Scotland could lead the way in this, and send a signal to the rest of the UK about how things could be done in a more humane society’ (Participant 1, woman, cousin, West Scotland).

DCRs were widely viewed as marking a step towards a less enforcement-oriented response to drug use, by prioritising harm reduction over criminal sanctions. Several participants drew parallels between drug policies and policies directed to other behavioural issues, such as unhealthy eating or alcohol use. They expressed frustration that DCRs remained controversial, partly because drug dependence was often viewed as a moral issue rather than a matter of health. By contrast, it was argued that failure to implement life-saving interventions for conditions such as cancer would not be publicly acceptable:

You wouldn’t have somebody with cancer going to accident and emergency, [they] wouldn’t be sent away to find some chemotherapy or something. They would be given treatment’ (Participant 13, woman, mother, location unknown).

Regardless of how far participants supported more general drug policy reform, there was a shared sense that DCRs represented one aspect of a wider change in the way drug harms, and policy responses, were framed: away from a focus on enforcement and eradication of supply, towards principles of safety, prevention, protection, and the recognition of trauma:

‘It’s about creating a safe space really isn’t it, so if we have a premise that drug treatment service should be about creating safety. And its experience of safety which sits at the heart of trauma-informed approaches and responses, then why aren’t we also thinking about the other environments […]. Why aren’t we creating whole system responses that have a DCR alongside the police using their disruption and distraction, alongside people providing therapeutic support and input?’ (Participant 3, woman, mother, North Scotland).

The language and terminology surrounding drug consumption rooms were seen as significant with regard to stigmatisation. Many felt that the term ‘drug consumption room’ could exacerbate stigma and potentially inflame relations with local communities. One family member thought the term ‘consumption’ could create the impression that a facility was being funded merely for the consumption of illicit substances. By contrast, another felt that adding the word ‘supervised’ risked implying that the person required surveillance and was, therefore, a problematic person. Several suggested that the term ‘drug’ could detract from the service aims by focusing on drugs rather than harm reduction, safety, or support. When presented with various options, such as ‘safer consumption sites’, ‘safer injection sites’, and ‘overdose prevention centres’, many stated that ‘overdose prevention centre’ was the least stigmatising name. Two family members suggested that the service should simply be called ‘hope’, since that was what it represented to family members, people who use drugs, and local communities.

However, others felt that what mattered most was clarity about the service function itself:

‘It’s not about making it look better. It’s about [saying] “a spade is a spade, a drug is a drug”. It’s what it is. Be honest about it’ (Participant 4, woman, mother, East Scotland).

In summary, family members believed that DCRs could provide a useful addition to the range of services that could be implemented to prevent and reduce DRDs and other harms. Although some level of prior activism was apparent, most family members appeared to have learned about the intervention through media reporting including reporting related to the activities of an unsanctioned mobile DCR that was active at the time of data collection. Some disagreement was evident with respect to the role that DCRs could play in recovery from drug problems but, in general, participants believed that the intervention could, or could have, helped their own family members, and that implementation in Scotland would symbolise a more supportive approach to responding to people experiencing drug-related harm which they welcomed.

## Discussion

This was a novel and timely study. While there is a small but growing literature on family members perspectives on drug policy [54, 55], we are not aware of any other research focused on family member views on DCR implementation. Family member perspectives are vital to developing a comprehensive picture of how drug policy issues are framed, and to understanding how interventions may affect people with this lived experience. Since we recruited a convenience sample, we cannot know the extent to which participant views reflect those of the wider population of affected family members. However, for those who did participate, we found that opinions on DCRs were motivated not only by their own lived experience but also by a combination of hearing personal accounts from others and accessing some forms of research evidence. UK media extensively reported on plans to introduce a DCR in Glasgow so this may have also been a source of information [28]. Many participants felt that the evidence supporting DCRs was convincing and ascribed the continuing controversy to stigma and a lack of public understanding of the underlying causes of harmful substance use. It was, in this respect, perceived as an issue of values as much as evidence. Public acceptability was seen as an ongoing barrier to implementation. Several participants felt research such as this could help address this through providing insights into the difficulties and pain family members experience, and speaking to how DCRs could help alleviate some of that worry by providing a safe and supportive environment for their loved ones.

For many interviewees, DCR implementation was viewed as representing not only a key policy development, but also a societal shift towards acceptance that their loved ones, and people who use drugs more widely, were deserving of care and safety. This is supported by qualitative research among people who used DCRs in Ottawa, Canada, which highlighted that the role of DCRs goes well beyond reducing the acute risk behaviours associated with drug-related morbidity and mortality [56]. Furthermore, for some family members, DCRs represented a symbolic shift towards the replacement of a criminal justice enforcement-led approach to drug harms with a ‘health first’, compassionate, and person-centred response. The potential for DCRs to not only prevent overdose but to provide a ‘gateway’ to sustained treatment and/or recovery services was widely acknowledged and considered vital. Participants placed considerable emphasis on this function of DCRs and saw it as a core aspect of them.

Participants did, nonetheless, express some concerns, most notably that DCRs might enable continuation of, rather than reduce, their loved ones’ drug use. This is somewhat aligned with the UK Government’s assertion that DCRs would ‘encourage’ drug use, yet reflected a more nuanced perspective on, and a divergence of views towards, the relationship between harm reduction and recovery in this context. This reflects wider tensions between harm reduction and abstinence-led approaches which can be a barrier to implementation of DCRs in Scotland and internationally. Family members interviewed in this study acknowledged these complexities and the potential role that DCRs could play in addressing both perceived and actual gaps between harm reduction and recovery or abstinence-focused services. They felt that evidence that DCRs can provide important links to other health and social services, including drug treatment and recovery services, was very important [7, 40, 41, 56]. Participants felt that the risk of DCRs facilitating or ‘enabling’ drug use could be significantly reduced by the provision of signposting and referral to wider treatment and support—as has been implemented in many international DCRs and was proposed for the Glasgow service model [57].

Language plays an important role in perpetuating stigma [58]. This applies not only to PWUD, but also the nomenclature of DCRs. The array of competing names for essentially the same service likely reflects the extent to which different providers, advocates, and stakeholders feel the relationship between description and political messaging needs to be handled. Empirical research from the USA has suggested that there is greater public support when the name used for these types of services emphasises the core goal of saving lives (e.g. overdose prevention sites), rather than implying the purpose is simply to facilitate the consumption of controlled drugs (e.g. supervised injection facility) [59, 60]. Importantly, however, some family members also felt that naming decisions should not only consider what would be acceptable to potential decisions-makers or local communities (e.g. removing terms such as ‘drug consumption’), but should also be sensitive to the need for the purpose of the sites to be clear to people who would potentially use the service.

Our interviews revealed that the more family members learned more about the role and function of DCRs the more supportive they became. We noted that there were shifts in perspective during some of the interviews as a direct consequence of having discussions about DCRs, and that there was an increase in support for DCRs as a consequence of the research interviews in some cases. However, our study does not allow us to identify whether support was a direct consequence of increased knowledge, or whether some of this learning was motivated by prior support. Nevertheless, we were able to identify some key events that had triggered awareness of DCRs in participants. Prominent among these was the establishment of an unsanctioned mobile overdose prevention van in Glasgow shortly prior to data collection starting which received substantial media coverage. Participants reported that this had raised awareness of DCRs and prompted discussion in family support networks and other groups. The overdose prevention van appeared to have created a shift in media coverage, increasing the volume of discussion around DCRs and presenting a lay ‘proof of concept’: demonstrating in concrete terms how such a facility works. Media coverage also presented the lived experience of both its founder and others using the facility to provide a humanising narrative which may also have shifted public perceptions [61]. Previous empirical work has suggested that public support for DCRs in Scotland was highest when factual information was presented alongside a sympathetic narrative of the impact of a DRD on families, and a pre-emptive refutation of common public concerns [62].

Relatedly, those who were strongly in favour of DCR implementation emphasised the need for both a general public education campaign and provision of relevant information about the role and functions of DCRs, to increase knowledge and awareness among other affected families and also the general public. Public awareness campaigns are currently being run in Scotland to raise awareness of DRDs and how to respond to an overdose, and to target the reduction of stigma experienced by people who use drugs [63, 64]. Findings generated in this study could be used to inform future campaigns.

Many of the participants in this study felt strongly that the voices of families affected by, supporting, or in relationships with people who use drugs, should play a prominent role in debates on reducing harm. This was not only because participants believed their experiences deserved to be heard in the policy debate, but because they felt they were uniquely able to tackle the stigma that characterised public discourse on drug use, harms, and policy. By expressing the lived experience of loving, supporting, and suffering alongside people whose drug use had become problematic, they felt they could show that this was an issue of compassion and care, not punishment and condemnation. Research on personal narratives and policy change supports this perception [39–41]. It is therefore possible that a greater public profile for family experiences would—alongside wider dissemination of information and evidence—increase public support for, or acceptance of, DCRs as a harm reduction intervention.

### Strengths and limitations

This study had a number of strengths and makes an important contribution to existing literature. In particular, while several previous studies have examined the views and levels of support for DCR from the perspective of people who use drugs, the general public, and other stakeholders such as community service providers and business owners [65], this is, to the best of our knowledge, the first study to examine the views of family members of those people experiencing problems with drugs. While data were collected from people all over Scotland, this was a convenience sample and so motivation to take part in the research may have been based on prior interest in the topic: participants with more supportive views of DCRs may have been more willing to take part. Despite our aspirational starting point of including a diversity of family members, our recruited sample was largely women and specifically mothers. However, while our sample may not be representative of the views held by other family members of people who use drugs, interviews were rich and insightful, and there was diversity in opinion within the sample on many key issues. The study was conducted during the COVID-19 pandemic which created pressures for everyone, not least for individuals already experiencing challenging circumstances. Those who were able to take part in the study very openly shared their views and experiences which we are very grateful for. Further research with a more diverse sample of individuals from this overlooked group is required.

## Conclusion

The findings from this novel study reveal strong support for DCR implementation among participants. This support was motivated by personal experience and the need for facilities that addressed the unique needs of families dealing with problematic drug use; by exposure to the narratives of other families going through similar experiences; and by engagement with wider research that demonstrated positive results. DCRs were seen as valuable not only for the primary goal of preventing overdoses and other acute risks associated with injecting, but for providing accessible and potentially attractive gateways to further support and treatment. While concerns about enabling drug use were voiced among some, these concerns appeared to be outweighed by a belief that the overall impact would be positive. DCRs were also viewed by family members as potentially contributing to a paradigm shift in attitudes to drug use, and by extension drug policy, which enabled a move away from focusing on criminalisation and punishment to a focus on compassion and person-centred care. In this respect, DCRs were seen as important for both individual and societal reasons: they provided both a service and signified a set of values. It was this set of values that, above all, unified participants in this study in their support for DCRs, and in their belief that the testimony of families could, and should, play a prominent role in the public debate. This set of values was underpinned and reinforced by the sense of urgency relating to Scotland’s excessive DRDs, and an appeal from family members to speed up moves towards implementation.

## Supplementary Information

**Additional file 1:** Interview topic guide.

## Data Availability

The data set that informed this paper is not available for wider sharing because participants did not provide their approval for this.

## Abbreviations

BBV: Blood-borne virus
COREQ: Consolidated Checklist for Reporting Qualitative Research
DCR: Drug Consumption Room
DDTF: Drug Deaths Taskforce
DRDs: Drug-Related Deaths
GP: General Practitioner
HIV: Human immunodeficiency virus
NHS: National Health Service
RAG: Research Advisory Group
