# Supplementary material for: ‘Why would we not want to keep everybody safe?’ The views of family members of people who use drugs on the implementation of drug consumption rooms in Scotland
Source: Harm Reduct J. 2022 Aug 29;19:99. doi: 10.1186/s12954-022-00679-5 (PMC9421633; doi:10.1186/s12954-022-00679-5)
Supplement: Supplementary file 1 — Additional file 1: Interview topic guide. [file 12954_2022_679_MOESM1_ESM.docx]

**Appendix 1 – Interview topic guide**

**Perceptions and attitudes of strategic decision-makers and affected families across Scotland towards Drug Consumption Rooms to prevent drug-related deaths**

**Project title:** Perceptions and attitudes of strategic decision-makers and affected families across Scotland towards Drug Consumption Rooms to prevent drug-related deaths

**Document:** Qualitative interview topic guide – family members

**Version:** 3.0 **Date:** 1 October 2020

**Preamble**

- Thank you for giving up your time and agreeing to participate.
- Confirmation of: the purpose of the interview (exploration of perceptions and attitudes of family members across Scotland towards Drug Consumption Rooms), about the research team and funding, explore the participation information sheet, voluntary nature and explicit use of data (confidentiality).
- Recording of interview and transcription.
- Outline structure of interview.

**Themes, questions and topics**

| **Broad topic area** | **Opening question** |
| --- | --- |
| Awareness of DCRs | How aware of DCRs are you?  Have you been aware of the DCR debates in Scotland, UK and beyond? |
| Problem definition | What do you think DCRs aim to do? |
| Perceptions and attitudes towards DCRs | What are your current views towards DCRs?  Should DCRs be implemented in Scotland (whether across the whole country or in certain areas) as part of the national/local response to drug-related deaths? |
| Consequences | What do you perceive the likely impact of DCRs on:   1. drug use, overdoses, and infectious diseases for people who use drugs 2. families like yourself, of people who use drugs? 3. criminal behaviour? 4. Immediate neighbourhood of any location? |
| Formation of views | Where has your understanding about DCRs come from, if you’re able to pinpoint that? |
| Understandings of DCRs in context of wider drug treatment | How do you understand DCRs in the wider context of drug treatment? |
| Barriers | We’re interested to know what barriers you see in terms of setting DCRs up in Scotland? |
| Facilitators | We’re interested to know what you think would help DCRs to be introduced in Scotland? |
| Summing up DCR views | Do you think your family member would use or want to use a DCR?  Would you support/encourage your family member to use a DCR? |

Additional questions where time allows

| Nomenclature | There are a variety of other terms used for DCRs, such as Safer Injecting Rooms, Overdose Prevention Centres, Supervised Injecting Centres. What do you think about these different names, if you have a view? | Do interviewees see some terms as more or less accurate, appropriate, convincing etc.?  Do they have a preferred name, or name they think should be used widely/consistently? |
| --- | --- | --- |
| Prioritisation of DCRs in the Scottish context | Should DCRs be prioritised as a key response to help reduce drug-related deaths in Scotland?  Where do DCRs sit within other approaches concerning drug-related deaths? | Importance of DCRs or not,  Other types of interventions and where they fit e.g. anything else more needed/more appropriate that would help to keep their loved one safe?  Is there anything else more pressing/more important that would help the situation, help their loved one? |
| Anything else | Knowing we were going to have a conversation about DCRs, is there anything else you have thought about or think we should hear on the subject? | Participants given the option to provide any further information that they think might be relevant. |

Thank you very much for your time and for what you have told me today. Debrief sheet and wind down conversation and signposting to support organisations if needed.
